# Factors Associated to Psychological Distress During the COVID-19 Pandemic Among Healthcare Workers in Ecuador

**DOI:** 10.3389/ijph.2022.1604626

**Published:** 2022-03-22

**Authors:** Carlos Ruiz-Frutos, Cristian Arturo Arias-Ulloa, Mónica Ortega-Moreno, Macarena Romero-Martín, Kenny F. Escobar-Segovia, Ingrid Adanaque-Bravo, Juan Gómez-Salgado

**Affiliations:** ^1^ Department of Sociology, Social Work and Public Health, Faculty of Labour Sciences, University of Huelva, Huelva, Spain; ^2^ Safety and Health Postgraduate Programme, Universidad Espíritu Santo, Guayaquil, Ecuador; ^3^ Department of Economy, University of Huelva, Huelva, Spain; ^4^ Department of Nursing, University of Huelva, Huelva, Spain; ^5^ Facultad de Ingeniería en Ciencias de la Tierra, Escuela Superior Politécnica del Litoral, Guayaquil, Ecuador; ^6^ Facultad de Ingeniería en Mecánica y Ciencias de la Producción, Escuela Superior Politécnica del Litoral, Guayaquil, Ecuador

**Keywords:** COVID-19, SARS-CoV-2, psychological distress, Ecuador, healthcare professionals

## Abstract

**Objective:** The global COVID-19 pandemic has challenged health systems. Healthcare professionals had to face harsh conditions that have caused psycho-emotional consequences. Ecuador has been one of the countries hit hardest by the pandemic in Latin America. The objective of this study was to analyse the levels of psychological distress among healthcare workers in Ecuador during the COVID-19 pandemic.

**Methods:** A cross-sectional descriptive study was conducted with a convenience sample of 1,056 healthcare professionals, assessing their psychological distress, physical symptoms of COVID-19, state of health, the preventive measures adopted, and the history of contact with people infected with the SARS-CoV2 virus.

**Results:** showed that 66.0% of the participants manifested psychological distress, with significantly higher levels in women with symptoms of COVID-19 and previous contact with infected people or objects (*p* < 0.001). However, adherence to preventive measures and perception of health were associated with less psychological distress (*p* < 0.001).

**Conclusions:** The importance of monitoring the mental health of healthcare workers during the COVID-19 pandemic was confirmed, having identified factors associated with the development of psychological distress among professionals in Ecuador.

## Introduction

The World Health Organization (WHO) declared the Sars-CoV-2 virus outbreak in early 2020 as a global public health emergency [1]. Although the outbreak was initially located in China, it quickly spread throughout Asia and the rest of the world. According to the WHO, as of the end of July 2021, there have been 190,770,507 confirmed cases of COVID-19 worldwide, of which 4,095,924 have died [2]. In April 2021, Mautong et al. published that in Ecuador, the first case of coronavirus was identified in February 2020, and after a few weeks, in the city of Guayaquil alone, the largest in the country, approximately 2,200 cases were reported, which corresponded to 70% of the cases in the country [3]. The number of confirmed cases is 476,065 and 21,953 deaths according to the WHO [2]. However, as Cevallos-Valdiviezo et al. pointed out, the actual figure could be much higher due to underestimation derived from delays in death records, the lack of enough test kits across the country, false-negative test results, or the incorrect attribution of COVID-19 deaths [4].

The spread of the pandemic is accelerating thanks to the fluidity of the international mobility of the population and the ease of transmission of the virus [5]. COVID-19 is transmitted through close contact with an infected person, through droplets or aerosols emitted by the infected person during respiratory activities such as talking, coughing, laughing, sneezing. The receptor can be contaminated by direct inspiration from an infected aerosol in the vicinity of a sick person or by touching a contaminated surface [6]. Many of the patients remain asymptomatic and are unaware of their spreading potential, which is the same as that of a patient with symptoms, something that aggravates and favours transmission [7].

Before the availability of the vaccine and in the absence of specific treatment, the most effective way to address the pandemic was preventive containment measures, hand hygiene, face mask, social distance, and isolation [8]. Despite these measures, the virus spread rapidly, collapsing health systems. The Sars-CoV-2 virus triggers a respiratory inflammatory crisis that, together with certain previous risk factors such as advanced age (≥65 years), male sex, hypertension, cardiovascular diseases, diabetes, COPD, and malignant neoplasms, causes the person to require hospitalisation, complex respiratory support, admission to the ICU and, in many cases, leads to death [9]. Patients with COVID-19 remain hospitalised between 5 and 29 days, and those admitted to the ICU have a mean stay between 1 and 3 weeks [10]. All this translates into a care overload that healthcare workers, especially those on the front line, have to face, working directly with COVID-19 patients. In addition to work overload and long hours, healthcare professionals have had to perform their work in very difficult physical and psychological conditions [11]. The complex situation of the pandemic forces them to work under the feeling of constant threat due to direct exposure to the pathogen itself, with a shortage, at times, of protective material, and also due to fear of transmitting the virus to family or friends that can lead to social isolation, or professional frustration at the loneliness and death of the coronavirus patients they care for [11–13].

These exceptional circumstances have an unavoidable impact on the mental health of healthcare workers. High levels of anxiety, depression, post-traumatic stress, insomnia, obsessive-compulsive symptoms, emotional disorders, and somatization among healthcare workers have been described during the COVID-19 pandemic [14–17]. These consequences are especially accentuated in nurses, women, involved in the diagnosis, treatment or care of patients with coronavirus [16]. During the pandemic, high levels of compassion fatigue and burnout have been reported among healthcare professionals and a significant decrease in compassion satisfaction [18, 19]. This burnout is not only conditioned by the overload of work and the psychological demands of the pandemic situation, but also by the feeling of threat the workers experience and the lack of social support [20]. The psychological and emotional load of healthcare workers has repercussions in the performance of their functions, by conditioning their capacity for attention, understanding, and decision-making [17]. It is therefore imperative to address the psychological needs of healthcare workers in order to effectively deal with the pandemic.

Although the health crisis caused by COVID-19 has been a challenge for all health systems in the world, for low and middle-income countries, which are currently in a situation of inadequate resources, the challenge of COVID-19 has led to a worsening of the health gap, especially in terms of mental health [21]. Ecuador has been one of the countries hardest hit by the pandemic with a mortality rate among the highest in Latin America [22], which reached 8.5%, although it was probably much higher as many people died from the virus but undiagnosed [23]. In March 2020, of the four geographical regions of Ecuador, the coast and the city of Guayaquil were the most seriously affected areas since they accounted for 82.57% of the confirmed cases of COVID-19 and coincided with an area that had already been seriously affected by dengue cases (84%) [24].

To cope with the advance of the pandemic, the Ecuadorian government established containment measures such as confinement, traffic and movement restrictions, and curfew [25]. However, these measures were implemented unevenly throughout the country due to the fact that there is no universal health coverage because of difficulties in communications and geographical. Some groups such as indigenous populations or refugees may find it more difficult to take preventive measures [26]. The response of the Ecuadorian public health system has been slow and insufficient. The high number of cases and deaths collapsed the system and evidenced its operational shortcomings and the absence of a strategic plan to contain the spread of the infection [23]. Given this scenario and with the country’s scarce health resources, the threat is particularly serious for Ecuadorian healthcare workers, who have to fight the pandemic in one of the countries with the highest number of cases and deaths per capita worldwide [24]. It is necessary to know the psychological impact of the pandemic on health staff due to the negative consequences it can trigger for the group itself, for the people affected, and for society in general given its leading role in addressing and containing the pandemic.

The objective of this study is to analyse the levels of psychological distress during the COVID-19 pandemic among healthcare workers in Ecuador.

## Methods

### Type of Study

The present study follows a quantitative, descriptive cross-sectional cohort design.

### Sample

A total of 1,235 questionnaires were collected and, after filtering, 179 questionnaires (14.49%) were eliminated for not having answered 99% of the questions, leaving 1,056 questionnaires. The variables that did not collect some responses were identified in the tables, indicating the number of responses collected. The final sample came from all the provinces of Ecuador, with highest percentages from the provinces of Pichincha (31.2%), Guayas (24.5%), Azuay (6.4%) and Tungurahua (6.3%). By region, Andean (66.1%), Coastal (29.7%), Amazon (4.1%), and Galápagos (0.1%). 64.96% were physicians, 12.59% were nurses and 22.44% were other healthcare professionals. 86.36% were involved in care activities in contact with the patients. The inclusion criteria for the research were the following: 1) being an active healthcare professional; 2) being over 18 years of age; 3) living in Ecuador during the pandemic generated by the SARS-CoV2 virus; and 4) accepting the informed consent located on the first page of the questionnaire prior to its start.

### Study Variables

In the present study, the psychological distress (PD) of healthcare professionals has been considered as a dependent variable, and the sociodemographic characteristics, the presence of physical symptoms of COVID-19 and health status, the preventive measures adopted, and the history of possible contacts with people infected by the SARS-CoV2 virus have been considered as independent variables.

### Measuring Instruments

For data collection, two instruments were used; one designed ad hoc for the assessment of independent variables, and the General Health Questionnaire (GHQ-12) for the assessment of PD.

The first instrument, developed by the authors of this study, was a self-administered questionnaire with items related to sociodemographic characteristics such as sex, age, marital status, level of education, type of work, children, pet, or having a disability. Participants were also asked whether they had symptoms associated with COVID-19 such as fever greater than 38°C, cough, headache, muscle pain, dizziness, diarrhoea, sore throat, coryza, chills, and breathing difficulties. The symptoms variable is analysed in the tables as continuous but was categorised in order to be considered in the CHAID analysis. To categorise the symptoms variable, the 25 and 75 percentiles have been considered as group limits. In relation to the state of health, participants were asked whether they had any chronic illnesses and, in addition, about medication, hospitalisation and medical care during the last 14 days. All these items were assessed with a dichotomous YES/NO answer question. The perception of one’s own health was assessed with a Likert scale of five options that ranged from very good to very bad. Questions about having had or believing to have had contact with any infected person or material, whether any family member or co-worker have been infected, and whether they had been performed a diagnostic test were included to assess contact with any infected person or material. Adherence to preventive measures was assessed with items on certain preventive behaviours such as hand washing, respiratory protocol, or social distancing. The participants assessed the frequency with which they had performed these measures through a Likert scale from 1 to 5, being 1 never and 5, always.

The second instrument used was the General Health Questionnaire (GHQ-12), which consists of 12 questions with 4 possible answers. The reliability of the results was revised, giving a Cronbach’s Alpha of 0.799. Analysis at the item level used all of the Likert values, while the scale sum was done based on a binomial scoring system. Each question can be categorised as 0 (if the answer is 1 or 2) and 1 (if the answer is 3 or 4). Values greater than or equal to 3 were estimated as a cut-off point, which represents that people with a score within the range at the cut-off point had psychological distress [27].

### Data Collection

Once the questionnaire was prepared, it was distributed online through the survey platform Qualtrics^®^. Invitation to participate was sent via email to official organisations of healthcare professional groups who were asked to facilitate their dissemination. In addition, participants were asked to distribute the questionnaire through their professional contacts and social networks, looking for the snowball phenomenon. Data collection took place between 2 April and 17 May 2020.

### Data Analysis

The frequencies, percentages, measurements of position and dispersion, depending on the type of variable, allowed a descriptive analysis of the data. Next, the chi-squared association test and the Student’s t-test for independent samples were used to contrast existence and differences or not of relationship with the dichotomised psychological distress variable. The Chi-squared automatic interaction detection (CHAID) method detected those variables most related to psychological distress through the creation of a classification tree. To do this, the chi-squared test of independence was used, selecting among the predictors the most significant ones. Among its advantages over alternatives, such as regression, we find that it is non-parametric, has no restrictions on independent variables and is based on the significance of the chi-squared statistic; it also avoids cross-analysis and identifies significant relationships between variables. The analyses were carried out with the statistical software SPSS 26.0 and R version 4.0.0.

### Ethical Considerations

Participants were previously informed about the purpose and means of the study. Participation in the study was entirely voluntary and posed no risk to the participants. Informed consent to participate in the study was obtained from the research subjects prior to study commencement. The questionnaires were anonymous and recorded in a confidential database that could only be accessed by the research team. This study has the favorable report of the Research Ethics Committee of Huelva, belonging to the Regional Ministry of Health of Andalusia (PI 036/20) and by the Ethics Committee of the University of Portoviejo, Ecuador (USGP-DI-049-2021).

## Results

### Sociodemographic Data

Of the 1,056 questionnaires that were finally analysed, there was a predominance of women (65.2%). 47.0% were 30 years of age or younger, 95.8% had university studies or higher, 57.7% were living without a partner, 48.6% with children, 65.6% were working as public employees, 56.3% were living with a pet, and 2.2% had a disability (Table 1).

**TABLE 1:** Association between sociodemographic variables and psychological distress during the pandemic (Ecuador, 2021).

	Healthcare staff (*N* = 1,056)				
	GHQ				
	N (%)	No (*N* = 359)	Yes (*N* = 697)	*χ* ^2^	*p*
Sex					
Male	368 (34.8)	44.6	55.4	28.118	**<0.001**
Female	688 (65.2)	28.3	71.7		
Age^a^ (*N* = 1,045)					
30 or younger	491 (47.0)	35.6	64.4	1.435	0.231
Older than 30	554 (53.0)	32.1	67.9		
Marital status					
With a partner	609 (57.7)	34.8	65.2	0.426	0.514
Without a partner	447 (42.3)	32.9	67.1		
Educational level					
Upper secondary school or lower	44 (4.2)	43.2	56.8	1.726	0.189
University or higher	1,012 (95.8)	33.6	66.4		
You are (*N* = 811)					
Independent	79 (9.7)	36.7	63.3	7.070	**0.029**
Public employee	532 (65.6)	31.2	68.8		
Worker for private company	200 (24.7)	41.5	58.5		
Children					
Yes	513 (48.6)	35.1	64.9	0.530	0.467
No	543 (51.4)	33.0	67.0		
Pet					
Yes	594 (56.3)	34.0	66.0	0.000	0.993
No	462 (43.8)	34.0	66.0		
Disability					
Yes	23 (2.2)	39.1	60.9	0.276	0.599
No	1,033 (97.8)	33.9	66.1		

a Grouped variable from median value. GHQ, General Health Questionnaire.

Statistically significant results are presented in bold.

### Psychological Distress

As shown in Table 2, the items that gave the highest mean ratings in PD were question 5 “Have you felt constantly overwhelmed and in tension?” (M = 2.81; SD = 0.88) and 7 “Have you been able to enjoy your normal daily activities?” (M = 2.69; SD = 0.87). On the contrary, the questions with the lowest values were number 10 “Have you lost confidence in yourself?” (M = 1.77; SD = 0.92) and 11 “Have you thought that you are a worthless person?” (M = 1.34; SD = 0.72). The overall mean score obtained on a total of a 12-point scale was 4.64 (SD = 3.47). Establishing a cut-off point for values greater than or equal to 3, the results showed that 66.0% of the study participants presented psychological distress (Table 2).

**TABLE 2:** Psychological distress: General Health Questionnaire GHQ-12 (Ecuador, 2021).

	Healthcare staff (N = 1,056)
Item	M (SD)
1. Have you been able to concentrate well on what you were doing?	2.50 (0.73)
2. Have your worries made you lose a lot of sleep?	2.66 (0.96)
3. Have you felt that you are playing a useful role in life?	1.81 (0.85)
4. Have you felt capable of making decisions?	1.94 (0.78)
5. Have you felt constantly overwhelmed and stressed?	2.81 (0.88)
6. Have you had the feeling that you cannot overcome your difficulties?	2.23 (0.93)
7. Have you been able to enjoy your normal daily activities?	2.69 (0.87)
8. Have you been able to adequately cope with problems?	2.27 (0.72)
9. Have you felt unhappy or depressed?	2.44 (0.96)
10. Have you lost confidence in yourself?	1.77 (0.92)
11. Have you thought that you are a worthless person?	1.34 (0.72)
12. Do you feel reasonably happy considering all the circumstances?	2.18 (0.74)
GHQ-12 (over 12 points)	4.64 (3.47)
Cut-off point ≥ 3	**N (%)**
Yes	697 (66.00)
No	359 (34.00)
α-Cronbach (healthcare staff)	0.799

### Sociodemographic and Psychological Distress Variables

Analysing the sociodemographic variables (Table 1) and their relationship with developing PD, with a cut-off point of GHQ ≥ 3, women showed a higher percentage of PD (71.7%) than men (55.4%), *p* < 0.001. Public employees had higher PD compared to employees of private or independent companies (*p* = 0.029). On the contrary, no statistically significant difference has been observed in the development of PD with regards to the variables age, educational level, living as a couple, having children, having a pet, or having a disability.

### Physical Symptoms and Psychological Distress

The highest percentages of the presence of physical symptoms (Table 3) resulted in headache (44.3%), coryza (27.7%), sore throat (25.3%), and muscle pain (21.9%). Regarding the association between presenting symptoms and generating PD, all of them showed a statistically significant difference except for fever, observing greater significance the symptoms of headache, dizziness, or sore throat, and being respiratory difficulty the one that scored a higher percentage of PD (84.4%). The number of symptoms was another variable associated with PD (*p* < 0.001).

**TABLE 3:** Association between physical symptoms and health and psychological distress during the pandemic (Ecuador, 2021).

		GHQ			
	N (%)	No	Yes	*χ* ^2^	*p*
Fever					
Yes	55 (5.2)	32.7	67.3	0.042	0.838
No	1,001 (94.8)	34.1	65.9		
Cough					
Yes	199 (18.8)	27.1	72.9	5.143	**0.023**
No	857 (81.2)	35.6	64.4		
Headache					
Yes	468 (44.3)	26.1	73.9	23.542	**<0.001**
No	588 (55.7)	40.3	59.7		
Myalgia					
Yes	231 (21.9)	26.0	74.0	8.480	**0.004**
No	825 (78.1)	36.2	63.8		
Dizziness					
Yes	124 (11.7)	20.2	79.8	11.985	**0.001**
No	932 (88.3)	35.8	64.2		
Diarrhoea					
Yes	134 (12.7)	23.1	76.9	8.070	**0.005**
No	922 (87.3)	35.6	64.4		
Sore throat					
Yes	267 (25.3)	25.8	74.2	10.587	**0.001**
No	789 (74.7)	36.8	63.2		
Coryza					
Yes	292 (27.7)	27.1	72.9	8.667	**0.003**
No	764 (72.3)	36.6	63.4		
Chills					
Yes	59 (5.6)	22.0	78.0	3.985	**0.046**
No	997 (94.4)	34.7	65.3		
Breathing difficulties					
Yes	45 (4.3)	15.6	84.4	7.123	**0.008**
No	1,011 (95.7)	34.8	65.2		
**Health**					
Perceived health					
Optimal	882 (83.5)	37.0	63.0	20.975	**<0.001**
Mediocre/ very bad	174 (16.5)	19.0	81.0		
Chronic illness					
Yes	180 (17.0)	30.6	69.4	1.145	0.285
No	876 (83.0)	34.7	65.3		
Currently taking medication					
Yes	226 (21.4)	26.1	73.9	7.977	**0.005**
No	830 (78.6)	36.1	63.9		
Hospitalised last 14 days					
Yes	11 (1.00)	27.3	72.7	0.224	0.636
No	1,045 (99.0)	34.1	65.9		
Medical care last 14 days					
Yes	103 (9.8)	29.1	70.9	1.206	0.272
No	953 (90.2)	34.5	65.5		
**Symptoms**					
	M (SD)			Statistical	*p*
N^o^ symptoms	1.77 (2.01)	1.33 (1.75)	2.00 (2.1)	−5.520	**<0.001**

Statistically significant results are presented in bold.

There was no association between having a chronic disease, having been hospitalised, or having received medical care during the last 14 days and developing PD, an association that was found among those who were taking medication at the time of the study (Table 3). 83.5% answered that they had an optimal health perception (very good or good), versus mediocre or lousy.

### Contact History and Psychological Distress

As can be seen in Table 4, 58.3% of the study participants reported having or not knowing whether they had had close contact with people confirmed to be infected with COVID-19, 55.1% had or did not know if they had had casual contact, and 63.8% said they had or did not know if they had had any contact with a person or material suspicious of being infected. All three assumptions were associated with symptoms of PD (*p* < 0.001). 75.1% of participants said that no member of the family had been infected, and 17.3% had been performed a diagnostic test. Neither of these cases was associated with PD (Table 4).

**TABLE 4:** Association between variables related with history of contacts and psychological distress during the pandemic (Ecuador, 2021).

		GHQ			
	N (%)	No	Yes	Statistical	*p*
Contact >15′ <2 m with infected person					
Yes, or does not know	616 (58.3)	29.9	70.1	11.217	**0.001**
No	440 (41.7)	39.8	60.2		
Casual contact with infected person					
Yes, or does not know	582 (55.1)	28.5	71.5	17.314	**<0.001**
No	474 (44.9)	40.7	59.3		
Any contact with person or material suspicious of being infected					
Yes, or does not know	674 (63.8)	30.0	70.0	13.458	**<0.001**
No	382 (36.2)	41.1	58.9		
Infected family member					
Yes, or does not know	263 (24.9)	30.4	69.6	1.998	0.157
No	793 (75.1)	35.2	64.8		
Having been performed diagnostic test					
Yes	183 (17.3)	35.0	65.0	0.094	0.759
No	873 (82.7)	33.8	66.2		

Statistically significant results are presented in bold.

### Preventive Measures and Psychological Distress During the Pandemic

**FIGURE 1:**
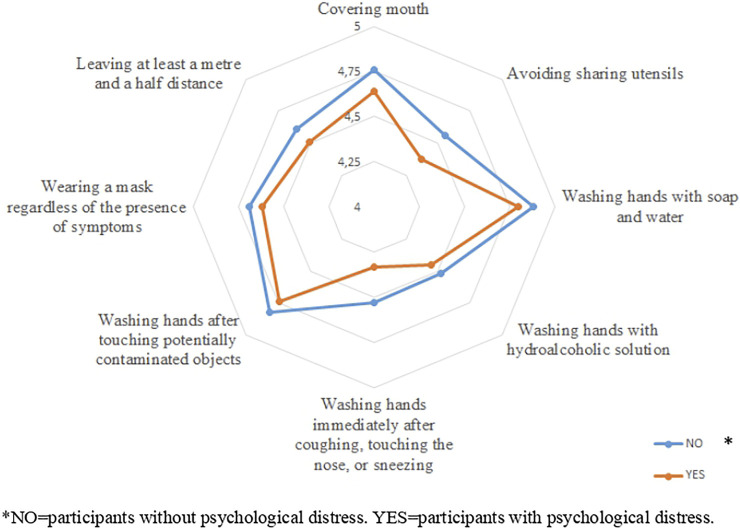
Mean values of preventive measures according to psychological distress (Ecuador, 2021).

The preventive measures that healthcare professionals scored a higher mean value were washing hands with soap and water (M = 4.83; SD = 0.45); washing hands after touching potentially contaminated objects (M = 4.77; SD = 0.52); covering the mouth when coughing or sneezing (M = 4.68; SD = 0.62); and wearing a mask (M = 4.65; SD = 0.75). The adherence to preventive measures was associated with PD, except in washing hands with hydroalcoholic solution and wearing a mask regardless of the presence of symptoms (Figure 1).

### Prediction of Psychological Distress During the Pandemic in Healthcare Professionals

**FIGURE 2:**
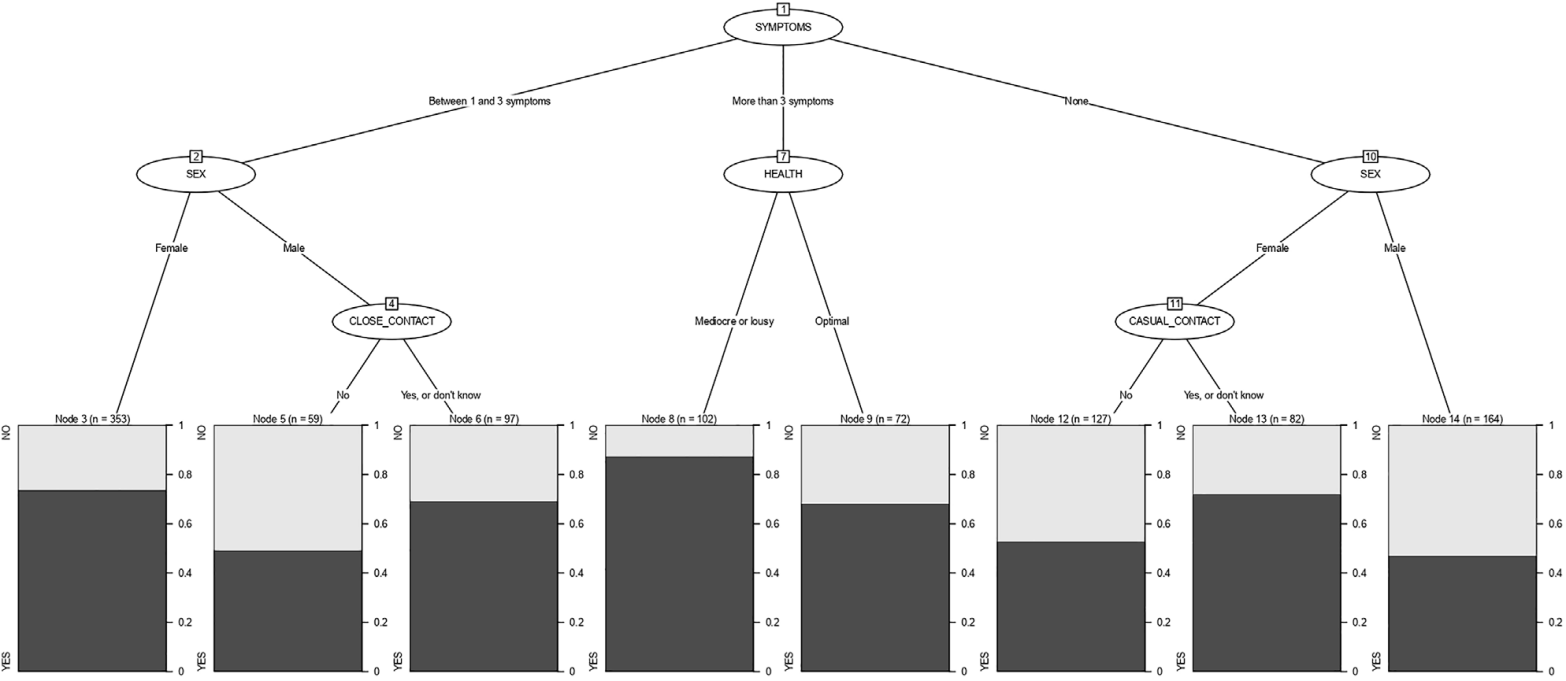
Segmentation tree displaying the level of psychological distress on the basis of sex, symptoms, contact with the virus and perceived health (Ecuador, 2021).

In the 1,056 cases studied, the CHAID method indicated the number of symptoms as the most significant variable related with psychological distress, distinguishing between without symptoms, one to three symptoms and more than three symptoms. For 35.3% of people without symptoms (373), men imply 47.0% of PD cases, as compared to women, whose proportion varies between 72.0% and 52.8% depending on whether or not there has been casual contact with someone whose infection has been confirmed. Among people with one, two, or three symptoms (509), sex was also a differentiating node and, as in the previous case, the number of cases with PD was higher in women, 73.7%, than in men. In the latter, the fact of having had or not close contact more than 15 min at less than 2 m with a person confirmed of infection made these percentages vary between 49.2% and 69.1%, respectively. The perception of health in the last 14 days was the differentiating node when there have been more than three symptoms (174 cases); if the perception is optimal, the percentage of distress was 68.1%, and reached 87.3% when the perception of health was mediocre or lousy (Figure 2).

## Discussion

The results of the study revealed a moderate-high level of psychological distress (66.0%) among healthcare professionals during the COVID-19 pandemic. These results coincide with those obtained by other international studies on anxiety, depression, and stress among healthcare professionals working on the front lines of the COVID-19 crisis [14, 28, 29]. Previous studies that included Ecuadorian healthcare professionals in their sample also obtained similar results in relation to the manifestations of stress (59.5%), anxiety (45.7%), depression (55.4%), and post-traumatic stress (70.2%) [5, 30]. However, our results were higher than those found by Mautong et al. in their study on the mental health of the Ecuadorian population during confinement, obtaining 17.7% of depression, 30.7% of anxiety, and 14.2% of stress [3]. This difference is consistent with previous studies that identified significantly higher levels of PD among healthcare professionals, as compared to the general population [31, 32]. In particular, distress has been described as especially high in front-line healthcare professionals versus those who do not work with COVID-19 patients [16, 19].

Some authors have suggested the lack of resources and protective equipment as a factor associated with healthcare workers’ anxiety in the face of the pandemic [33, 34]. In Ecuador, the health system’s response to the coronavirus outbreak was insufficient, especially regarding healthcare human resources and the provision of personal protective equipment [23]. The results found by Martín-Delgado et al. described a shortage of personal protective material manifested by 70% of healthcare workers in Brazil, Colombia, and Ecuador, especially of suits, masks, and face shields, even in areas where aerosol-generating procedures were performed [35]. To address the lack of material and technical resources, Ecuador requested international assistance and eventually had to accept donations from the Pan-American Health Organization (PAHO) of protective equipment for front-line workers [23]. The deficiencies not only affected the supply of equipment, but also the training on its correct use, since 51.5% of Ecuadorian healthcare professionals admit not having received training on the use of personal protective equipment [35]. However, Zhang et al. study on organisational support for healthcare workers in Peru, Ecuador, and Bolivia did not identify risk control and protection as a predictor of professionals’ anxiety [36].

On the other hand, it has been pointed out that being informed about the health crisis through official sources with verified data is associated with fewer PD [37] and that a greater number of daily hours obtaining information from different sources increases PD in non-healthcare workers [38]. Ecuadorian healthcare professionals have said they are unaware of the protocols for the care of COVID-19 patients [35]. This lack of knowledge could explain the high levels of PD found in our study. Chen et al. conducted a study on the COVID-19 beliefs of healthcare workers in Ecuador and identified conspiracy theories regarding the origin of the virus. Professionals who believed the virus was intentionally developed in a laboratory were more likely to have an anxiety disorder [39]. The study by Bates et al. on the Ecuadorian general population, despite identifying good knowledge about COVID-19, concluded that this was not enough to motivate a change of attitude towards the pandemic and confidence in overcoming it [40].

According to our results, women have greater psychological distress than men, which resembles previous similar studies [30, 31, 41]. Similarly, in the present study, the presence of COVID-19 symptoms and the history of contacts with infected people was associated with higher levels of PD and taking preventive measures against COVID-19 implied a lower decline of the mental health of healthcare workers. Similar results were obtained by Wang et al. in their study on psychological responses during the first stage of the pandemic in China. According to these authors, participants with symptoms such as chills, myalgia, cough, dizziness, rhinitis, sore throat, and shortness of breath had higher levels of anxiety, depression, and post-traumatic stress [42]. In addition, according to the results found by Alkhamees et al. from a similar study, not experiencing any of the symptoms listed above was significantly associated with lower PD scores [43]. It is striking that, as in previous studies, the least frequent symptoms were fever and respiratory difficulties, given that COVID-19 is an inflammatory respiratory disease.

As our results show, contact with infected people or material has previously been identified as an influential factor for the psychological impact on healthcare professionals [44]. Healthcare professionals working in the front line, at greater risk of infection by close contact with COVID-19 patients, have reported greater psychological deterioration than others [16, 19]. It has been identified that these professionals are more likely to suffer from anxiety, depression, and insomnia [16, 45] and have more secondary traumatization [31]. Exposure to risk and threat perception leads professionals to feel more vulnerable and, as a result, suffer more PD [46, 47]. The lack of protective measures has been associated with higher levels of anxiety and depression among healthcare professionals [48].

In Ecuador, in the sociocultural context in which this study was developed, the population has suffered a greater psychological exhaustion than other countries, which confirms our results [5]. The Ecuadorian coast was considered the region most affected by COVID-19, with the highest number of cases [24] and significantly higher levels of anxiety and depression [22]. An international study conducted in Ecuador, Chile, Colombia and Spain, revealed that Ecuadorians, although they perceived the health crisis with severity, showed less adherence to health recommendations and reported lower levels of awareness of their health status, as compared to other countries [30]. The psychological impact has had consequences for the health of the population in relation to their eating habits, with changes in meal times or increased intake and a worsening of sleep quality [49]. However, according to Bermerjo-Martins et al. [30] in relation to self-care, the elderly and women are generally more involved in self-care activities and adopt healthier daily routines. The crisis has been particularly severe for healthcare professionals, who, in addition to the exposure to risk and fear of contagion of their relatives, have endured an overload of work in difficult conditions, under psychological and emotional pressure. Despite working in this harsh scenario, healthcare professionals in Ecuador have manifested high levels of work engagement and compassion satisfaction, which describes them as a positively involved and committed group despite the difficulties [50]. Initiatives have been proposed that promote coping strategies and self-care to maintain the psycho-emotional balance of these workers and enable them to play their role in the fight against the pandemic [41]. In the Ecuadorian healthcare environment, it has been identified that exercising, maintaining daily routines, and staying informed about COVID-19, but limiting it to 1 h a day, is associated with better mental health [22].

### Limitations

As limitations to the present study, convenience sampling should be recognised, as it does not guarantee that it is representative of the study population, so some caution is recommended in the generalisation of the results. When an online data collection is carried out, the territorial distribution of the sample is not homogeneous. As mentioned, the highest incidence of cases is concentrated in the coastal area and the city of Guayaquil, so care overload is more intense in these areas and its impact on healthcare professionals could be expected to be geographically uneven. The data of the study were collected during the first phase of the pandemic and may have changed in the following phases, so a new collection has been planned with those participants who have voluntarily accepted, which will allow to know their evolution. At the end of the questionnaire, participants were asked whether they were interested in repeating the questionnaire after 6 months to know if there were any modifications. In order to do so, they had to indicate an email address to be contacted.

### Conclusion

It was evidenced that the level of psychological distress for health staff in Ecuador was 66% of the sample analysed, with women presenting greater distress as compared to men. The presence of psychological distress was revealed to be associated with the number of symptoms of COVID-19, each of them except fever, and especially headache, with taking medication during the last 14 days, and having had contact with infected people or material. The better perception of health and adherence to preventive measures was associated with lower psychological distress, conditioned by availability of these preventive measures and their mandatory use in the workplace.

The results of this study are a contribution to the knowledge of the state of health of the Ecuadorian healthcare team fighting against COVID-19. Monitoring the mental health of healthcare workers is imperative to implement an adequate response to the COVID-19 pandemic. Further similar work is needed to allow a more complete diagnosis of the psycho-emotional state of Ecuadorian healthcare workers, identifying the factors associated with these alterations in order to monitor their mental integrity and design support and coping strategies that help them maintain the psycho-emotional balance necessary to continue exercising their essential role in the fight against the pandemic.
