# Corrigendum: Factors Associated to Psychological Distress During the COVID-19 Pandemic Among Healthcare Workers in Ecuador

**DOI:** 10.3389/ijph.2022.1604996

**Published:** 2022-06-21

**Authors:** Carlos Ruiz-Frutos, Cristian Arturo Arias-Ulloa, Mónica Ortega-Moreno, Macarena Romero-Martín, Kenny F. Escobar-Segovia, Ingrid Adanaque-Bravo, Juan Gómez-Salgado

**Affiliations:** ^1^ Department of Sociology, Social Work and Public Health, Faculty of Labour Sciences, University of Huelva, Huelva, Spain; ^2^ Safety and Health Postgraduate Programme, Universidad Espíritu Santo, Guayaquil, Ecuador; ^3^ Department of Economy, University of Huelva, Huelva, Spain; ^4^ Department of Nursing, University of Huelva, Huelva, Spain; ^5^ Facultad de Ingeniería en Ciencias de la Tierra, Escuela Superior Politécnica del Litoral, Guayaquil, Ecuador; ^6^ Facultad de Ingeniería en Mecánica y Ciencias del la Producción, Escuela Superior Politécnica del Litoral, Guayaquil, Ecuador

**Keywords:** COVID-19, SARS-CoV-2, psychological distress, Ecuador, healthcare professionals

In the original article, there was a mistake in Figure 1 as published. The legend incorrectly stated “*NO=participants with psychological distress. YES=participants without psychological distress”. The correct version is “*NO=participants without psychological distress. YES=participants with psychological distress.” The color of this legend has been changed from red to black.

**FIGURE 1:**
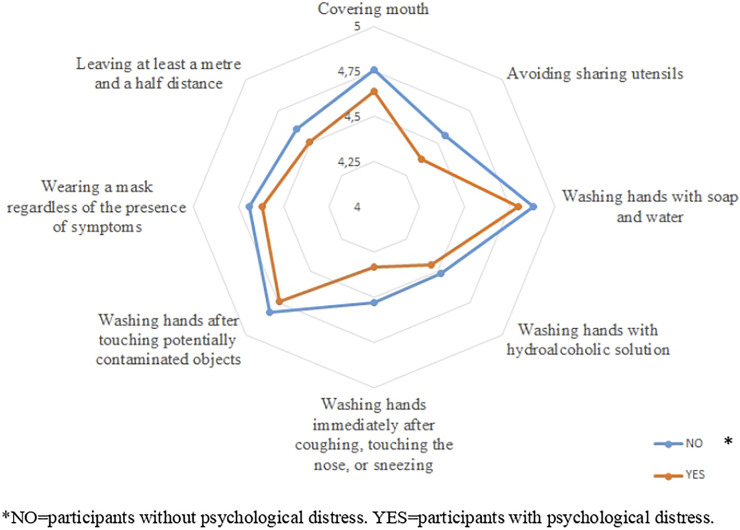
Mean values of preventive measures according to psychological distress (**Ecuador, 2021**).

The authors apologize for this error and state that this does not change the scientific conclusions of the article in any way. The original article has been updated.

